# Review of the Variations of the Superficial Veins of the Neck

**DOI:** 10.7759/cureus.2826

**Published:** 2018-06-18

**Authors:** Dominic Dalip, Joe Iwanaga, Marios Loukas, Rod J Oskouian, R. Shane Tubbs

**Affiliations:** 1 Seattle Science Foundation, Seattle, USA; 2 Anatomical Sciences, St. George's University, St. George's, GRD; 3 Neurosurgery, Swedish Neuroscience Institute, Seattle, USA; 4 Neurosurgery, Seattle Science Foundation, Seattle, USA

**Keywords:** external jugular, vein, superficial, internal jugular, thyroid vein

## Abstract

The venous drainage of the neck can be characterized into superficial or deep. Superficial drainage refers to the venous drainage of the subcutaneous tissues, which are drained by the anterior and external jugular veins (EJVs). The brain, face, and neck structures are mainly drained by the internal jugular vein (IJV). The superficial veins are found deep to the platysma muscle while the deep veins are found encased in the carotid sheath. The junction of the retromandibular vein and the posterior auricular vein usually form the EJV, which continues along to drain into the subclavian vein. The anterior jugular vein is usually formed by the submandibular veins, travels downward anterior to the sternocleidomastoid muscle (SCM), and drains either into the EJV or the subclavian vein. Other superficial veins of the neck to consider are the superior, middle, and inferior thyroid veins. The superior thyroid and middle thyroid veins drain into the IJV whereas the inferior thyroid vein usually drains into the brachiocephalic veins.

## Introduction and background

The external jugular vein (EJV) is the preferred vein when performing a central venous catheterization. The other options are the internal jugular vein (IJV) and the basilic, subclavian, and femoral veins through subcutaneous access [1]. Variations in the superficial veins of the neck are important to be aware of when performing neck, vascular, or any other surgery in their region, in the hope of preventing unintentional injury [2-3]. The aim of this paper is to review the anomalies and variations of the major superficial veins of the neck.

## Review

External jugular vein

**Figure 1:**
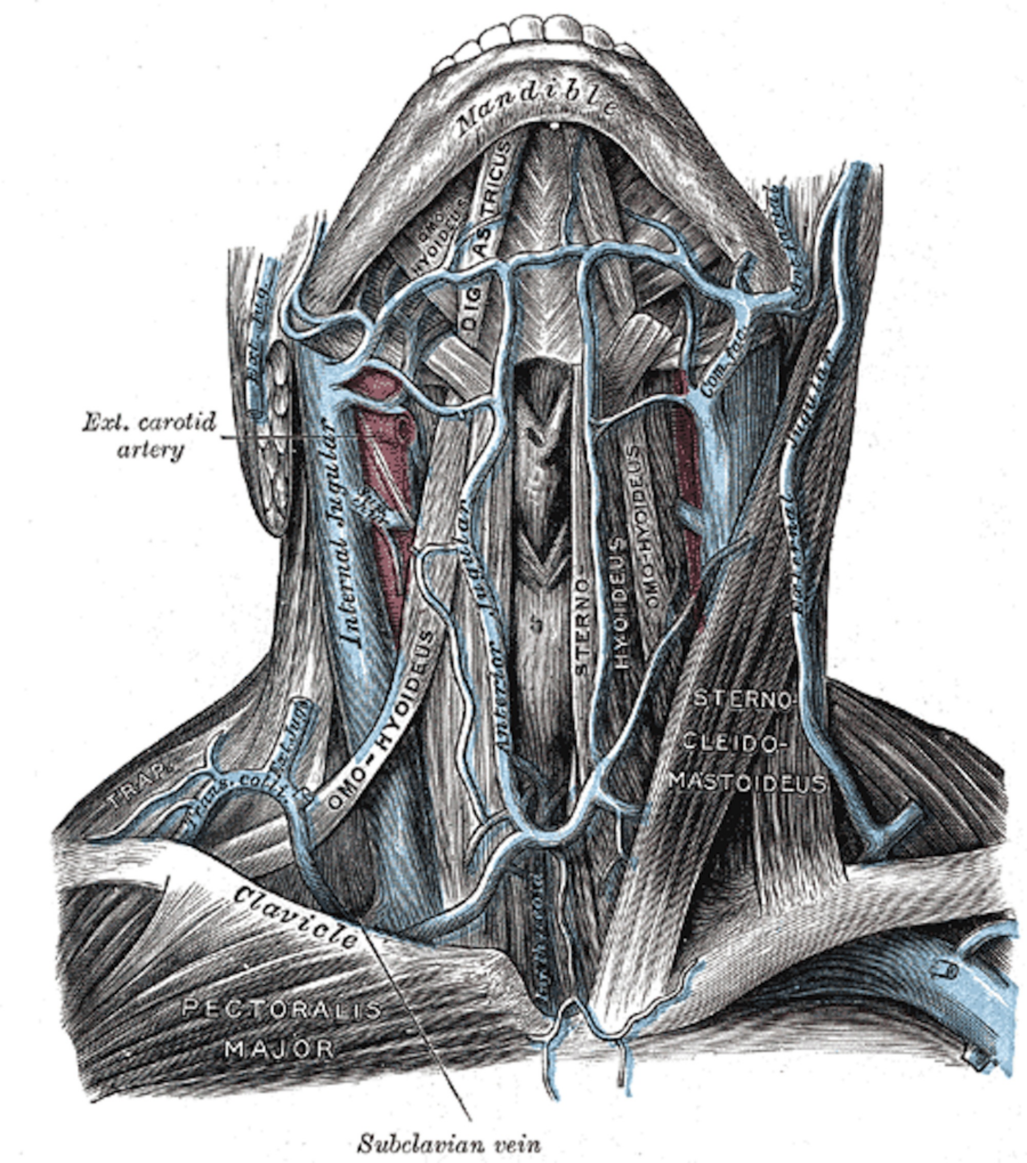
The external jugular vein formed by the retromandibular and facial veins and forming the external jugular vein, which then drains into the internal jugular vein

**Figure 2:**
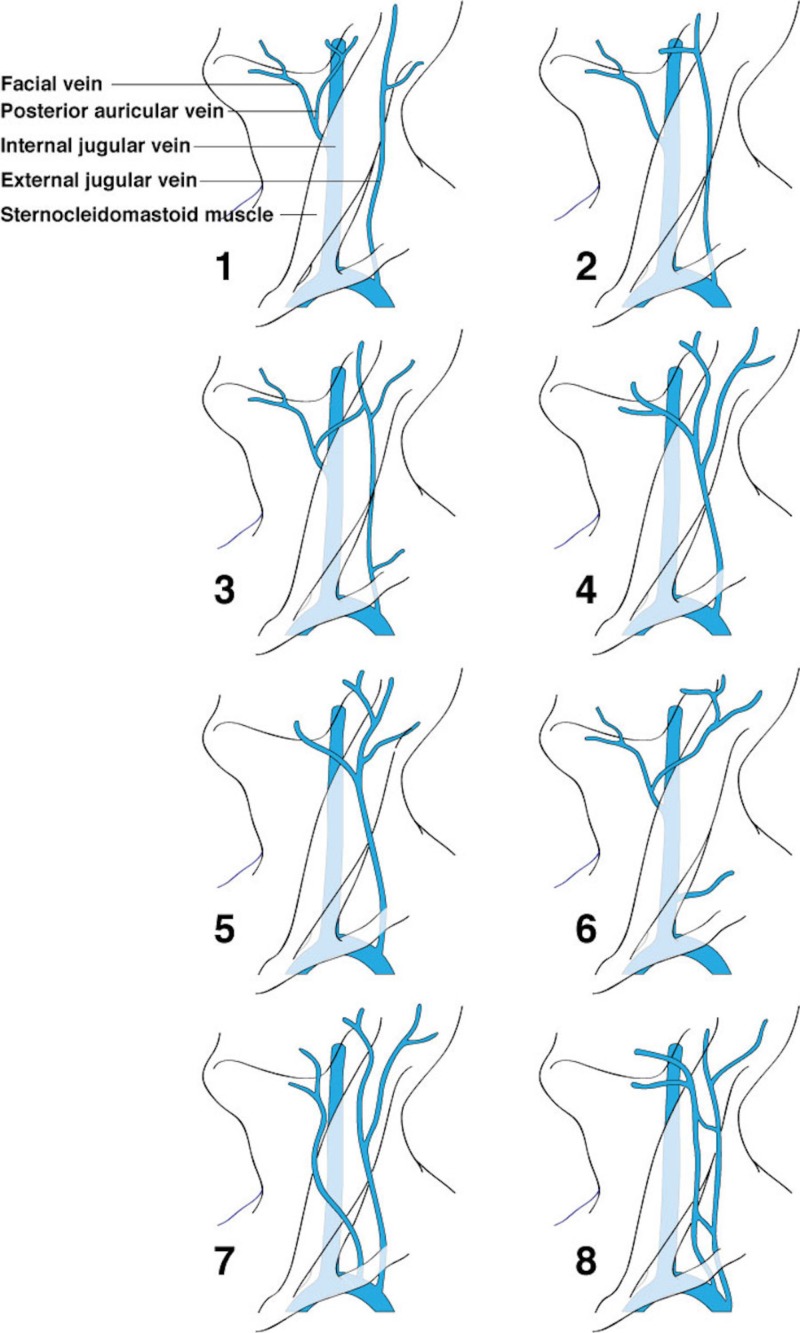
Variations in the formation of the external jugular vein

The junction of the posterior division of the retromandibular vein and the posterior auricular vein usually form the EJV that then continues to drain into the subclavian vein [3-6]. The EJV is sometimes absent ipsilaterally or bilaterally, which means that the veins forming the EJV usually drain into the IJV [7-8]. The EJV usually receives distribution from the transverse cervical vein, the anterior jugular vein, the suprascapular vein, and the superficial cervical vein (Figure 1). The EJV flowed into the jugulo-subclavian venous confluence in 60% of cases, 36% into the subclavian vein at a distance from its junction with the IJV, and 4% into the trunk of the IJV [5,9]. The EJV was seen duplicated before penetrating deep fascia, in the middle third near the posterior border of the sternocleidomastoid muscle [10]. Additionally, a double EJV emerging from the parotid gland as two independent veins has been described [11]. The presence of three right-sided EJVs running in a parallel fashion and draining into the right subclavian vein in a male cadaveric specimen was reported [8]. Moreover, in this cadaver, there were two anterior jugular veins that drained into the right brachiocephalic vein. Figure 2 summarizes the more frequent variations of the EJV.

Vein of Kocher (posterior external jugular vein)

There can be many variations with respect to the venous drainage of the neck. One such variation is a vein originating in the occipital region, usually a tributary of the common facial vein, which travels obliquely along the anterior border of the sternocleidomastoid muscle (SCM) and eventually drains into the IJV, middle part of the EJV, jugular venous arch, or brachiocephalic vein [2,8]. The posterosuperior superficial muscles and skin are drained by this vein. This vein is called the vein of Kocher or the posterior external jugular vein. This vein can be the same size or larger than the IJV, which makes it easy to misidentify [2].

Anterior jugular vein

The anterior jugular veins can have variations such as one vein running in the midline of the neck, which is termed the median cervical vein [1,8]. Another variation manifests as two parallel anterior jugular vessels, one opening into the external jugular vein and one into the transverse cervical vein. Sometimes, one anterior jugular vein may open into the external jugular vein and the other into the subclavian vein but can also give a large communicating branch to the anterior division of the IJV [6,12]. A case was reported where the left anterior jugular vein drained into the terminal portion of the IJV and the right anterior jugular vein drained into the confluence of the subclavian vein and the IJV [13]. The submandibular venous arch is formed when the anterior jugular joins the EJV [13].

Jugular venous arch

The jugular venous arch is usually formed by the two anterior jugular veins, just above the sternum, which travels downward along the midline between the pretracheal and superficial layers of the cervical fascia, i.e., in the space of Burns. The jugular venous arch either drains into the subclavian or the EJV. When performing a low tracheostomy, this vessel may be encountered. One variation is that the jugular venous arch can receive tributaries from small branches of the inferior thyroid tributaries [1,14].

Inferior thyroid vein

**Figure 3:**
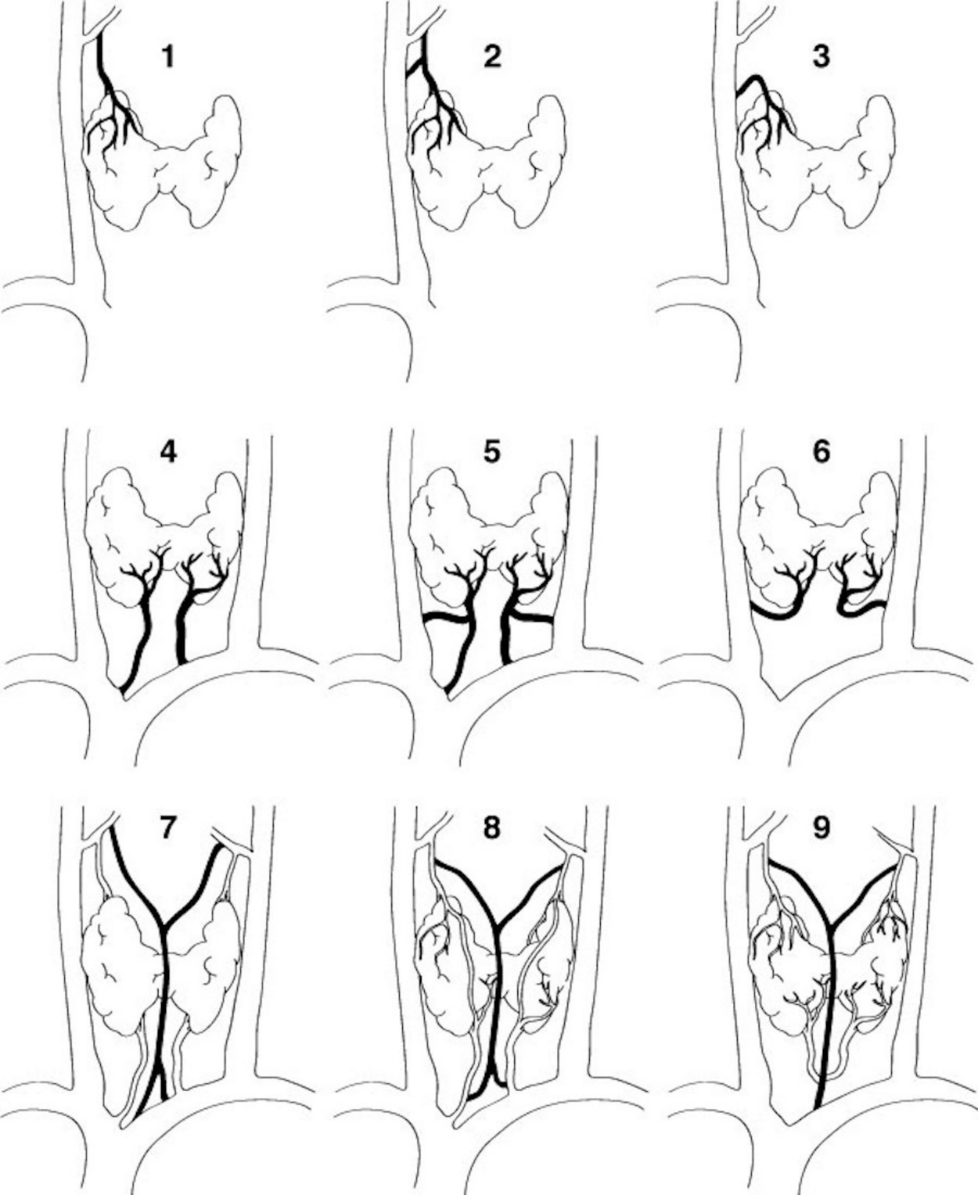
Various combinations of drainage of the superior, middle, and inferior thyroid veins Labels 1-3 demonstrate superior thyroid vein variations; 4-6 shows inferior thyroid vein variations; images 7-9 show variant combinations of the superior and inferior thyroid veins. Note the “median” thyroid vein formed in 7, 8, and 9.

The inferior thyroid veins usually drain into the brachiocephalic veins and then into the superior vena cava. Sometimes, they may drain into the IJV and anastomose with the thymic veins [15]. In 41% of cases, the inferior thyroid veins on each side joined the ipsilateral brachiocephalic vein separately. In 35% of cases, the inferior thyroid veins first formed a common trunk before joining the left brachiocephalic vein [16]. Figure 3 summarizes many of the variations of the inferior thyroid veins.

Superior thyroid vein

In one study, the superior thyroid vein was found bilaterally (83.3%), with its termination (87.1%) into the IJV (97.2%)—either isolated (29.4%) or with other veins such as the lingual vein (52.1%) [17]. Additionally, anastomoses are commonly seen between the superior thyroid vein and the common facial vein [5].

Middle thyroid vein

The variations in the middle thyroid vein are important when performing surgical procedures involving the thyroid and parathyroid glands or near the recurrent laryngeal nerve. This vein usually crosses the common carotid artery anteriorly and eventually drains into the IJV. Sometimes, however, it can also drain into the brachiocephalic vein. The middle thyroid vein was found in 38% of dissected lobes and 62% of operated patients, and in 80% of cases, originated in the mid-region of the thyroid gland [18]. One fetus was found to have a middle thyroid vein that drained into the vertebral vein [19].

As apparent from this review, the superficial veins of the neck can be quite varied. Therefore, a good understanding of the variant anatomy of the neck region and its related vasculature is important for those performing invasive and minimally invasive procedures in this area [20-25]. Complications can be minimized with a good understanding of both normal and anomalous vascular anatomy.

## Conclusions

Physicians should be made aware of the presence of variations of the superficial veins of the neck. Being mindful of these variations allows clinicians and surgeons performing neck, vascular, or reconstructive surgery the ability to prevent unintended injury during procedures.
